# Phenotypic and functional analysis of lymphocytes infiltrating osteolytic tumors: use as a possible therapeutic approach of osteosarcoma

**DOI:** 10.1186/1471-2407-5-123

**Published:** 2005-09-27

**Authors:** S Théoleyre, K Mori, B Cherrier, N Passuti, F Gouin, F Rédini, D Heymann

**Affiliations:** 1 INSERM ERI 7 – EA 3822; Physiopathologie de la Résorption Osseuse et Thérapie des Tumeurs Osseuses Primitives. Faculté de Médecine, 1 rue Gaston Veil, 44035 Nantes cedex 1, France

## Abstract

**Background:**

Osteosarcoma is the most common type of primary bone tumor. The use of aggressive chemotherapy has drastically improved the prognosis of the patients with non-metastatic osteosarcomas, however the prognosis of the patients with metastasis is still very poor. Then, new and more effective treatments for curing osteosarcoma, such as immunotherapy are needed. Tumor-infiltrating lymphocytes (TIL) have been involved in the control of tumor development and already assessed with success for the treatment of several cancers including melanoma. While TIL represent a fascinating therapeutic approach in numerous malignant pathologies, there is few report concerning adult bone-associated tumors including osteosarcoma.

**Methods:**

Human TIL were isolated and characterized (phenotype, lytic activity) from twenty-seven patients with bone-associated tumors (osteosarcoma, Ewing's sarcoma, giant cell tumor, chondrosarcoma, plasmocytoma and bone metastases). Similar experiments were performed using rat osteosarcoma model.

**Results:**

While TIL with a main CD4^+ ^profile were easily isolated from most of the tumor samples, only TIL extracted from osteosarcoma were cytotoxic against allogeneic tumor cells. In all cases, TIL lytic activity was significantly higher compared to autologous peripheral blood leukocytes. Similar data were observed in rat osteosarcoma model where TIL were characterized by a main CD4^+ ^profile and high lytic activity against allogeneic and autologous tumor cells. Moreover, rat TIL expansion was not accompanied by refractoriness to further activation stimulus mainly by tumor antigens.

**Conclusion:**

These results demonstrated that TIL therapy could be a very efficient strategy for the treatment of adult osteosarcoma.

## Background

Primary or secondary malignant bone tumors represent a major therapeutic challenge in medical oncology. Despite efficiency of conventional treatments by chemo- and radiotherapy, long-term outcome of the patients suffering from malignant bone tumors remains poor. Among them, osteosarcoma is the most frequent primary bone tumor. Indeed, the current strategy for the treatment of high-grade osteosarcoma is based on neo-adjuvant chemotherapy, delayed en-bloc wide resection and adjuvant chemotherapy adapted to the histologic profile of the tumor tissues removed during surgery [1]. While marked improvements in surgery and the development of different regimens of multidrug chemotherapy over the past 25 years, the survival remains around 55 to 70% after 5 years [2,3]. Furthermore, the prognosis is worse in the patients with non-extremities localization, advancing age, radio-induced osteosarcoma and those arising from Paget's disease of bone, representing 40% of the entire osteosarcoma population. In addition, the patients with metastatic osteosarcoma at the time of diagnosis have poor survival statistics (30% at 5 years). All these findings suggest necessity of establishing new therapeutic strategies to improve the overall rate of survival, especially in high-risk sub-groups.

Among the potential new therapeutic strategies, immunotherapies are based on the up-regulation of the immune response in tumor-bearing host. Two therapeutic approaches can be distinguished: (i) active immunotherapies that elicit immune response against tumor cells in tumor-bearing host (including pulsed dendritic cells and cytokine treatments), (ii) passive or adoptive immunotherapies consist on the administration of *ex vivo*-expanded tumor-specific cytotoxic immune cells represented by T lymphocytes. The identification of tumor-specific lymphocytes has resulted in new therapeutic strategies based on mounting a sustained and effective anti-tumor immune response [4,5]. It is theorized that the infiltrating lymphoid represents a selected population of cells which have preferentially migrated to the tumor secondary to an immune response. These T lymphocytes termed tumor-infiltrating lymphocytes (TIL) are considered to be more specific in their immunological reactivity to tumor cells than the non-infiltrating lymphocytes [6]. However, if the role of TIL has not been clearly defined, TIL could recognized as a reactional mechanism against tumor development. Moreover, their reduced response in the tumor tissue may be due to the suppressive influence under the tumor microenvironment. In this context, TIL have been identified in numerous neoplasia, such as melanoma [5-9], various carcinomas [10-17], myeloma [18], pediatric tumors [19] and sarcomas [20-22] and the therapeutic relevance of these TIL has been also documented in both animal models and clinical trials [23-28]. Unfortunatly, up to date, very few data is available on the phenotypic and functional characterization of TIL isolated from adult bone-associated tumors.

Because, *in vitro *studies on bone tumor infiltrating T lymphocytes are lacking, the goal of the present study is to characterize the TIL derived from 27 adult human bone-associated tumors as well as autologous peripheral blood leukocytes (PBL) at the phenotypic and functional levels. The therapeutic potential of TIL was then discussed for osteosarcoma.

## Methods

### 1) Patients

Twenty-seven bone-associated tumors [6 osteosarcomas, 2 Ewing's sarcomas, 2 chondrosarcomas, 7 giant cell tumors (GCTs), 2 plasmocytomas, 4 bone metastases (2 from kidney origin, 2 form unknown origin) and 4 other pathologies (1 chondromyxoid fibroma, 1 fibrous dysplasia, 1 chordoma, 1 undifferentiated sarcoma)] from 27 patients [12 women (38 + 17.8 years, range: 17–75) and 15 men (47.9 + 19.2) years, range: 16–75)] were included in the present study. All patients were treated in the Department of Orthopedic Surgery of Nantes University Hospital (France) between September 2004 and June 2005. Patients' characteristics were summarized in Table 1 ("Additional file 1"). Written informed consent was obtained before each patient was included in the study.

### 2) Cell lines

The rat osteosarcoma (UMR106), human osteosarcoma (MG63, SaOS2), B lymphoma (Daudi) and leukemia (K562) cell lines were purchased from the American Tissue Cell Collection (USA). The rat osteosarcoma ROS 17/2.8 cell line was a kind gift from Pr HJ Donahue (Penn State University, USA). The tumor cell lines were culturedin RPMI 1640 (Invitrogen, France) supplemented with 10% foetal calf serum (FCS) (Hyclone, Perbio, France). The primary osteosarcoma was isolated from small tumor fragment of rat osteosarcoma model described above. Briefly, small tumor fragments were digested by 1 mg/mL collagenase A (Boehringer Manheim) for 2 hours. The cell suspensions obtained were then washed three times with PBS and cultured in RPMI 1640 supplemented with 10% FCS. The medium was changed twice a week and the adherent osteosarcoma cells were harvested using a 0.05% trypsin- 0.02% EDTA solution and subjected to the study.

### 3) Osteosarcoma rat model

The osteosarcoma was initially induced by a local injection of colloidal radioactive ^144^Cerium in rats [29]. The tumor can be re-grafted and maintained *in vivo *for many months; otherwise fragments were frozen until re-utilization. This rat osteosarcoma model used in the present study mimics the human pathology in terms of tumor growth, bone involvements and lung metastases [30,31].

### 4) Peripheral blood leukocytes (PBL) preparation and tumor-infiltrating lymphocytes (TIL) extraction

Both human and rat PBL were isolated from heparinized peripheral blood after Ficoll-Hypaque (Eurobio, France) gradient centrifugation. The human and rat PBL were respectively cultured in RPMI 1640 supplemented with 10% FCS and 300 UI/ml IL-2 (Eurocetus, France) for 21 days and X-VIVO 15 supplemented with 900 UI/ml IL-2 for 14 days. Their phenotype and cytotoxic activity were then analyzed according to the procedure described below.

Human tumor specimens were directly received from the operating room under sterile condition, minced with scissors into 1 mm^3 ^fragments and cultured immediately in RPMI 1640 (Invitrogen) supplemented with 10% FCS (Hyclone), 300 UI/ml IL-2 (Eurocetus) and antibiotics mixture (100 UI/mL penicillin and 100 μg/mL streptomycin). Half of medium was changed twice a week and TIL were cultured for 21 days.

Rat tumor specimens were obtained from the model described above, minced with scissors into 9 mm^3 ^fragments and subjected enzymatic digestion using 2 mg/mL collagenase and 50 UI/mL hyaluronidase (Sigma-Aldrich, France) for 2 hours. The cell suspension (10^6 ^cell/mL) was filtrated using a 70 μm cell strainer. Then, enriched by Ficoll-Hypaque gradient centrifugation and cultured in X-VIVO 15 serum free-medium supplemented with 900 UI/mL IL-2 and antibiotics mixture (100 UI/mL penicillin and 100 μg/mL streptomycin). Half of medium was changed twice a week and TIL were cultured for 14 days. Both PBL and TIL were maintained at 37°C in a 5% CO_2 _humidified atmosphere.

### 5) Cytotoxicity assay

Target cells (UMR106, MG63, SaOS2, Daudi, K562, ROS17/2.8 and primary autologous rat osteosarcoma cells) were labeled with 10 μCi of Na_2 _^51^CrO_4 _(Amersham, UK) for 1 hour at 37°C. Then, target cells were washed three times with fresh culture medium. Lymphocytes (TIL or PBL) were added at a different target/effector ratio (T/E) of 1/12.5, 1/25 and 1/50. The plate was then incubated for 4 hours at 37°C, and 100 μL of supernatant were haversted for counting in a γ-counter. Maximal and spontenous ^51^Cr-releases were determined by incubating the target cells with 100 μL of detergent (Triton 1% in distillated water) or 100 μL of culture medium, respectively. Spontaneous release (SR) never exceeded 10% of maximal release (MR). The percentage of specific chromium release was calculated according to the following formula: [(experimental release-SR)/(MR-SR)] × 100%].

### 6) Flow cytometric analysis

For phenotyping, lymphocytes (TIL or PBL) were stained with conjugated monoclonal antibodies against CD3 (PE), CD4 (APC), CD8 (perCP), CD20 (FITC), CD45RA (FITC), CD161 (PE). Monoclonal antibodies were purchased from Becton Dickinson (France) (CD3, CD4, CD8 and CD20) and from Serotec (France) (CD161 and CD45RA). Cell suspensions were then analyzed on a Becton Dickinson fluorescence-activated cell sorter. Ten thousand scatter-gated cells were analyzed in each sample and fluorescence profiles of the cells were determined with logarithmic signal amplifiers.

### 7) Statistical analysis

All experiments were performed in triplicate. The mean and SD was calculated for all conditions and compared by an ANOVA test. Differences relative to a probability of two-tailed *P *< 0.05 were considered significant

## Results

### 1) Human TIL can be extracted from various human tumor biopsies and cultured

**Figure 1:**
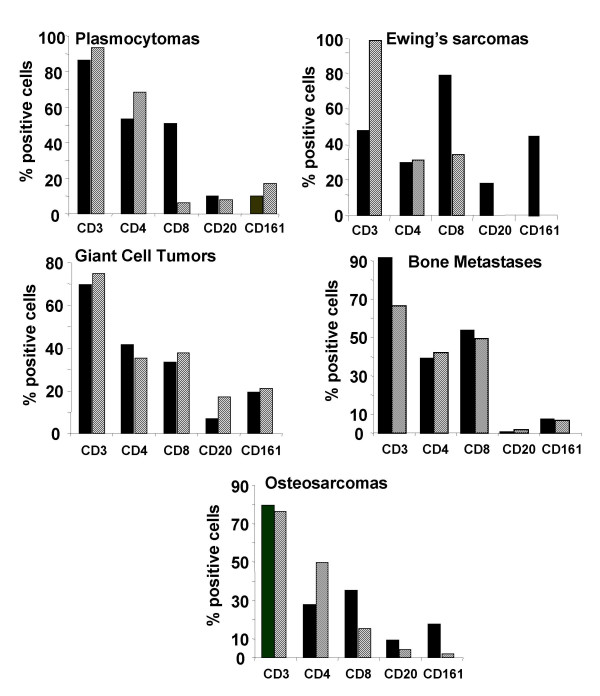
**Phenotypic analysis of TIL isolated from human bone associated tumors compared with the phenotype of autologous PBL. **TIL were isolated from tumor biopsies and cultured for 21 days in RPMI 1640 supplemented with 10% FCS and 300 UI/ml IL-2. Autologous PBL were isolated from peripheral blood samples after gradient density centrifugation and cultured in the same conditions for 21 days. After the indicated culture period, the phenotype of TIL (hatched bars) and PBL (dark bars) were analyzed by flow cytometry. The percentages of positive cells are given as median value. Plasmocytoma, n = 2; Ewing's sarcoma, n = 2; giant cell tumor, n = 7; bone metastase, n = 4; osteosarcoma, n= 6.

TIL were able to isolate successfully from small tumor fragments (wet weight < 5 g) immediately after the biopsy except for chondrosarcoma, fibrous dysplasia and 50% of bone metastases (Table 1) and maintained for 3 weeks in the presence of 300 UI/ml IL-2 for flow cytometric analyses. Most of cultured TIL were mainly composed of CD3^+ ^(range: 65%–99%) (Figure 1, hatched bars). TIL from plasmocytomas showed preferentially CD4^+ ^profile (median: 64.4%) with minor CD8^+ ^profile (median: 6.4%). Similarly, TIL from osteosarcomas were predominantly CD4^+ ^(median: 49.9%) with second highest CD8^+ ^sub-population (median: 15.5%). TIL isolated from Ewing's sarcomas, GCTs and bone metastases are characterized by almost same amount of CD4^+ ^and CD8^+ ^populations (around 50%) (Figure 1). A minor amount of CD20^+ ^and CD161^+ ^cells were observed in all TIL without correlation with tumor histology.

To compare PBL and TIL expanded from the same patients, phenotypic analyses of PBL were also performed in all included cases (Figure 1, dark bars). The amount of CD8^+ ^TIL was lower in plasmocytoma, Ewing's sarcoma and osteosarcoma TIL compared to that of PBL (respectively 88%, 58%, and 57%). A high amount of natural killer (NK) CD161^+ ^(43%) cells were detected in PBL from Ewing's sarcoma-bearing patients in contrast to the others.

### 2) Human TIL isolated from osteosarcomas exert cytotoxic activity against allogeneic cell lines

**Figure 2:**
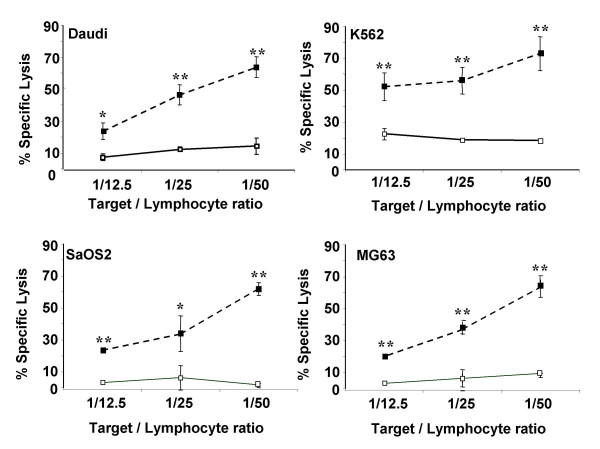
**Comparison of cytotoxic activity between TIL isolated from human osteosarcomas and autologous PBL. **TIL and autologous PBL were isolated from human osteosarcomas (n = 6). After 21 days of culture in RPMI 1640 supplemented with 10% FCS and 300 UI/ml IL-2, TIL and autologous PBL cytotoxic activities were determined using ^51^Cr-released assays. The cytotoxicity was measured against allogeneic cell lines including Daudi, K562 cell lines and two osteosarcoma cell lines, high (SaOS2) and low (MG63) osteoblastic differentiated osteosarcoma. ** p < 0.01; *** p < 0.001, TIL versus PBL cytotoxicity.

To determine the cytotoxic activity of expanded TIL against allogeneic cell lines, ^51^Cr-released assays were carried out. The lytic patterns of TIL against Daudi and K562 cell lines that reflect LAK and NK activity, and against two allogeneic osteosarcoma cell lines SaOS2 and MG63, which exhibit respectively high and low osteoblastic differentiation state were assessed. TIL amplified from plastocytomas, Ewing's sarcomas and GCTs did not show any lytic activity against all allogeneic cell lines tested (data not shown). However, TIL extracted from osteosarcoma biopsies were significantly cytotoxic against all cell lines analyzed compared to matched autologous PBL (p < 0.01) (Figure 2). The cytotoxic profiles against K562 and the other cell lines were different along with T/E ratio. Indeed, the magnitude of TIL cytotoxicity against K562 cells varied from 50% to 70% respectively for a ratio T/E of 1/12.5 to 1/50. The magnitude of TIL lytic activity against Daudi, SaOS2 and MG63 reached 20% for a ratio T/E of 1/12.5 to 65% for a ratio T/E of 1/50. In all osteosarcomas analyzed, autologous PBL were weakly cytotoxic against the four allogeneic cell lines, and no dose-response was observed in autologous PBL cytotoxic activity (Figure 2).

### 3) TIL isolated from rat osteosarcoma showed a similar phenotype and lytic pattern to human TIL

**Figure 3:**
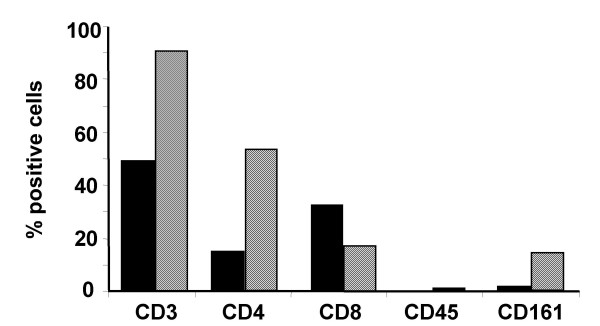
**Comparative analysis of TIL and autologous PBL phenotype isolated from rat osteosarcoma. **TIL were isolated from rat osteosarcoma biopsies (n = 8) by enzymatic treatment and cultured for 14 days in X-VIVO 15 supplemented with 900 UI/ml IL-2. Autologous PBL were isolated from peripheral blood samples after gradient density centrifugation and cultured in the same conditions for 14 days. After the culture period, the phenotype of TIL (hatched bars) and PBL (dark bars) were analyzed by flow cytometry. The percentages of positive cells are given as median value.

TIL expanded from rat tumor biopsies revealed same phenotype as those of human osteosarcoma. Namely, rat TIL were 90% CD3^+^, 50% CD4^+ ^and 18% CD8^+ ^in median value (Figure 3, hatched bars). Autologous PBL had an inverse CD4^+^/CD8^+ ^ratio with 16% CD4^+ ^and 32% CD8^+ ^cells in median value (Figure 3, dark bars).

**Figure 4:**
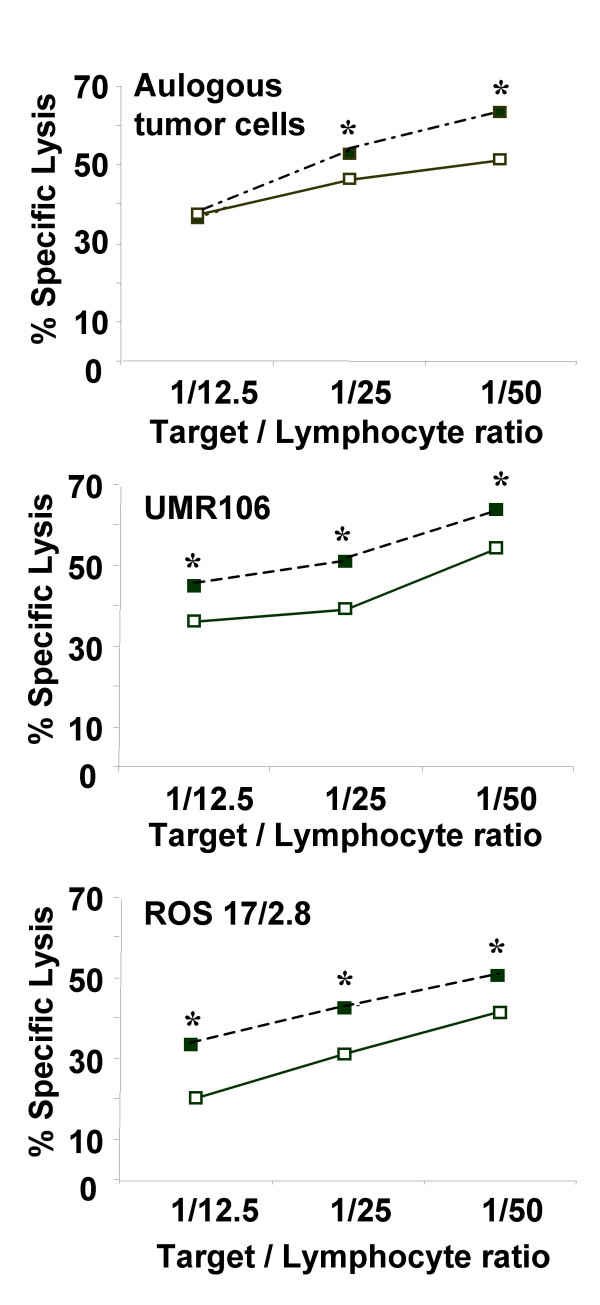
**Comparison of cytotoxic activity between TIL isolated from rat osteosarcomas and autologous PBL. **TIL were isolated from rat osteosarcoma biopsies (n = 8). After 14 days of culture in X-VIVO 15 supplemented with 900 UI/ml IL-2, TIL and autologous PBL cytotoxic activities were determined using ^51^Cr-released assays. The cytotoxicity was measured against autologous rat osteosarcoma cells and allogeneic rat osteosarcoma cell lines including UMR 106 and ROS 17/2.8. * p < 0.05, TIL versus PBL cytotoxicity.

^51^Cr-released assays also disclosed the similar lytic pattern of rat TIL against autologous and allogeneic tumor cell lines (UMR 106, ROS 17/2.8) to human TIL (Figure 4). Moreover, this lytic activity was higher against autologous tumor cells and UMR 106 (63% T/E of 1/50) compared to ROS 17/2.8 cells (48% T/E of 1/50) (Figure 4).

**Figure 5:**
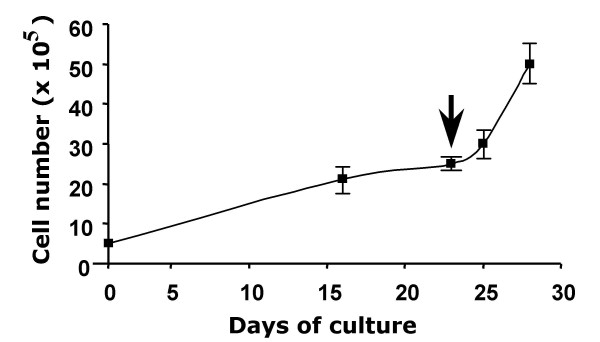
**Proliferation profile of TIL isolated from rat osteosarcoma. **These data are representative of three independent tumors. TIL isolated from rat osteosarcoma biopsies by enzymatic treatment were cultured at 5 × 10^5 ^TIL per well (6-multiwell plate) for 28 days in X-VIVO 15 supplemented with 900 UI/ml IL-2. At day 23 (arrow), 20 × 10^3 ^irradiated (75 grays) autologous osteosarcoma cells were added in each well. TIL were manually numbered using the trypan blue staining.

To elucidate the capability of autologous tumor cells to stimulate the proliferation of rat TIL, expanded TIL were cultured with irradiated tumor cells. Figure 5 showed the kinetic expansion of rat TIL without tumor antigens during the first 23 days and with irradiated tumor cells in the following term. TIL proliferation was characterized by gradual expansion (more than 4-fold) in the first 3 weeks culture and rapid proliferation reached 10-fold number within following five days culture (Figure 5). TIL expanded from rat osteosarcoma were very sensitive to the tumor antigens expressed by autologous tumor cells. These data demonstrated that TIL expanded from osteosarcoma were very sensitive to the tumor antigens expressed by autologous tumor cells.

## Discussion

The present data demonstrated that TIL could be isolated from the most of adult bone-associated tumors studied (osteosarcomas, Ewing's sarcomas, GCTs, plasmocytomas) except for chondrosarcoma, fibrous dysplasia and 50% of bone metastases; while only TIL extracted from osteosarcomas demonstrated a lytic activity against allogeneic tumor cell lines. Moreover, TIL extracted from osteosarcoma biopsies were significantly cytotoxic against all cell lines analyzed compared to matched autologous PBL. These findings demonstrating the interest of expanded TIL to target osteosarcoma cells.

Lymphocytes localized in a tumor foci could represent a tumor-specific response of T lymphocytes associated for one part to a non-specific component due to the inflammatory infiltrate. In this context, the adoptive transfer of specific-cytotoxic T cells has been already shown to induce tumor rejection in several animal tumor models [32,33]. Although isolation and characterization of TIL have been successfully performed in human malignant tumors [5-21], few data is available in the literature about bone-associated tumors except for pediatric tumors [34,35]. The presence of T lymphocytes in human osteosarcoma tissues was previously studied by Trieb *et al*. by immunohistochemical studies [36]. Phenotypic analyses have revealed that these infiltrating lymphocytes into osteosarcoma were 95% CD3^+ ^and 68% CD8^+ ^[36]. Rivoltini L *et al*. have also analyzed the phenotype of TIL in 37 pediatric tumors, including 12 osteosarcomas and demonstrated CD8^+ ^predominancy [34]. The latter also revealed that the lytic pattern of TIL against both allogeneic and autologous tumor cells were variable depend on the histology. Rivoltini L *et al*. concluded, in 1992, that TIL extracted from pediatric tumors were difficult to expand at levels required for immunotherapy, however they could be envisaged [34]. In our study, the phenotype of TIL isolated from adult osteosarcomas are very similar in all cases (two third of CD4^+ ^and one third of CD8^+^) and autologous PBL were weakly cytotoxic against the four allogeneic cell lines tested. This discrepancy between TIL and PBL could be explained by the homing processus of the tumor-specific T lymphocytes to the tumor foci. Indeed, recently, Haanen *et al *investigated the presence of TIL and circulating Tumor Associated antigens(TAA)-T cells before and after autologous tumor cell vaccination in metastatic melanoma patients [35]. They demonstrated that in none of the patients, TAA-specific T cells were found both in tumor tissue and circulating blood at the same time. No significant changes in the frequency and specificity circulating TAA-specific T were found during the treatment period in all patients while inside melanoma tissue, TAA-specific TIL could be detected in 75% of tumor samples analyzed. These data suggested that a possible homing phenomenon of the tumor-specific T cell population to the tumor site could contribute to the effectiveness of antitumor immunity.

An experiment rat osteosarcoma model mimicking the human pathology was used to elucidate the availability of TIL in an autologous system. TIL extracted from rat tumor biopsies demonstrated similar phenotype to human osteosarcoma TIL (predominantly CD4^+^) and exhibit a highly cytotoxic activity against autologous tumor cells. The predominance of CD4^+ ^TIL obtained from human and rat biopsies contrasts partially to the data reported by Topalian *et al*. [37]. Indeed, these authors showed a correlation between CD8^+ ^lymphocytes and high cytotoxic activity. However, the role of the CD4^+ ^T lymphocyte sub-populations on the CD8^+ ^TIL activity remains to be clearly defined. The CD8^+ ^T cell survival and activity against the most of the tumor is increased by CD4^+ ^T helper cells (CD4^+^CD25^-^) and downregulated by CD4^+ ^T regulator cells (CD4^+^CD25^+^) [38-41]. The CD4^+^CD25^+ ^T cells maintain an microenvironment in the tumor sites that conceal the immunogenicity of tumor to permit progressive growth of antigenic tumors. In such cases, the suppression (*in vivo *or *ex vivo *from TIL extracted) of the T regulator cells represents a possible design of immunotherapeutic approach. In contrast to these data, the intestinal tumors which are strongly associated with an inflammatory microenvironment regress after injection of CD4^+^CD25^+ ^T regulator cells [42]. The overall published papers raise the possibility of broader functions for regulatory lymphocytes in prevention and treatment of human cancers. Thus, CD4^+ ^predominance in TIL population could not be prejudicial to the success of T lymphocyte-based immunotherapy but could be closely related to their CD25^+ ^(low or high), CD25^- ^status.

The lytic activity exhibited by rat osteosarcoma TIL is lower against ROS 17/2.8 than against UMR 106 and autologous tumor cells, suggesting some common tumor antigens shared by tumor cells. In contrast to the results of Rivoltini *et al*. [34], the low proliferation rate of rat TIL could be easily re-stimulated by autologous tumor antigens (Figure 5). These data therefore revealed that stimulation by tumor antigens could be a more appropriate technique to expand TIL for their use immunotherapy.

In the past decade, several reports underlined the capability of cellular therapy for osteosarcoma. The identification of human tumor antigens has opened new areas of antigen-specific cancer immunotherapy specifically targeting these antigens [6]. Indeed, several tumor antigens such as melanoma-associated antigen (MAGE) [43], squamous cell carcinoma antigen recognized by T cells (SART) 1 [44], SART3 [45] and papillomavirus binding factor [46] are expressed in osteosarcoma and provided the rationale to develop cellular therapies for this pathology. SART3 is highly expressed in osteosarcoma [45]. Moreover, the SART3-derived peptides were able to induce HLA-A2-restricted and tumor-specific cytotoxic T lymphocytes [45]. These data strengthened the potential use of the SART3-derived peptides for specific immunotherapy of HLA-A2^+ ^patients suffering from osteosarcoma. Taken together with the prevalence of HLA-A24 [46], this strategy could be applicable for approximately 60% of patients with osteosarcoma. Furthermore, no severe adverse response associated with peptides administration, and a significant up-modulation of the cellular immune response against tumor cells in clinical trial using SART3-derived peptides in HLA-A24^+ ^patients with colon cancer [47] encourages further application of this strategy for osteosarcoma. More recently, Trieb *et al*. demonstrated that Hsp 72 is involved in the interaction between T lymphocytes and osteosarcoma cells expressing Hsp72 [48]. The cytotoxic potential of these lymphocytes might lead to an increased immune response leading to the rejection of the osteosarcoma [48]. In light of the complementary animal experiments, cytotoxic lymphocytes recognizing specific tumor antigens appear to be a more effective therapeutic strategy than polyclonal TIL-based immunotherapy. Combined TIL with above strategies therefore might be more effective to achieve desired results.

## Conclusion

The results of the present report reveal that TIL exhibiting a high cytotoxic activity can be easily isolated from adult osteosarcomas. The poor prognosis of osteosarcoma must lead to develop new therapeutic approaches, especially in high sub-group such as adult osteosarcomas. TIL therapy is one of the hopeful strategies contribute to answer this intention.

## Abbreviations

Tumor-infiltrating lymphocytes (TIL)

## Competing interests

The author(s) declare that they have no competing interests.

## Authors' contributions

TS and M K contributed equally to this work. TS, MK and CB and carried out the *in vitro *experiments (TIL extraction, phenotypic and functional analyses); PN and GF coordinated the patient healthcare included in the study and collected human samples. FR carried out and coordinated the animal studies. HD conceived of the study, participated in its design and coordination, and has written the manuscript. All authors read and approved the final manuscript.

## Pre-publication history

The pre-publication history for this paper can be accessed here:



## Supplementary Material

Additional File 1 **Table 1: Panel of patients studied. **27 patients treated in the Department of Orthopedic Surgery of Nantes University Hospital (France) between September 2004 and June 2005, were included in the present study. The Table summarize the patients' characteristics included. Click here for file
