# Supplementary material for: Phenotypic and functional analysis of lymphocytes infiltrating osteolytic tumors: use as a possible therapeutic approach of osteosarcoma
Source: BMC Cancer. 2005 Sep 27;5:123. doi: 10.1186/1471-2407-5-123 (PMC1262697; doi:10.1186/1471-2407-5-123)
Supplement: Additional File 1 — Table 1: Panel of patients studied. 27 patients treated in the Department of Orthopedic Surgery of Nantes University Hospital (France) between September 2004 and June 2005, were included in the present study. The Table summarize the patients' characteristics included. [file 1471-2407-5-123-S1.doc]

# Table 1. Panel of patients studied

| Patients | Sex | Age | Diagnosis | Localisation | TIL obtention |
| --- | --- | --- | --- | --- | --- |
| 1 | F | 29 | osteosarcoma | femur | + |
| 2 | M | 68 | osteosarcoma | humerus | + |
| 3 | F | 57 | osteosarcoma | sacrum | + |
| 4 | F | 17 | osteosarcoma | tibia | + |
| 5 | M | 28 | osteosarcoma | femur | + |
| 6 | M | 24 | osteosarcoma | pelvis | + |
| 7 | M | 16 | Ewing’s sarcoma | scapula | + |
| 8 | F | 19 | Ewing’s sarcoma | femur | + |
| 9 | M | 40 | chondrosarcoma | sacrum | - |
| 10 | M | 37 | chondrosarcoma | humerus | - |
| 11 | M | 59 | giant cell tumor | tibia | + |
| 12 | F | 39 | giant cell tumor | femur | + |
| 13 | M | 45 | giant cell tumor | humerus | + |
| 14 | F | 29 | giant cell tumor | knee | + |
| 15 | F | 29 | giant cell tumor | femur | + |
| 16 | M | 22 | giant cell tumor | femur | + |
| 17 | F | 21 | giant cell tumor | tibia | + |
| 18 | F | 44 | chondromyxoid fibroma | sternum | + |
| 19 | M | 63 | fibrous dysplasia | pelvis | - |
| 20 | M | 75 | chordoma | spine | + |
| 21 | F | 62 | Undifferentiated sarcoma | thigh | + |
| 22 | M | 59 | plasmocytoma | rib | + |
| 23 | F | 59 | plasmocytoma | rib | + |
| 24 | M | 49 | Bone metastase (kidney origin) | sacrum | + |
| 25 | M | 67 | Bone metastase (kidney origin) | humerus | - |
| 26 | M | 66 | Bone metastase (unknown origin) | collarbone | - |
| 27 | F | 75 | Bone metastase (unknown origin) | pubis | + |
